# Dietary carbohydrate quality and risk of breast cancer among women

**DOI:** 10.1186/s12937-021-00752-y

**Published:** 2021-11-26

**Authors:** Bahareh Sasanfar, Fatemeh Toorang, Elham Mohebbi, Kazem Zendehdel, Leila Azadbakht

**Affiliations:** 1 Department of Nutrition, School of Public Health, Shahid Sadoughi University of Medical Sciences, Yazd, Iran; 2 Cancer Research Center, Cancer Institute of Iran, Tehran University of Medical Sciences, P.O.Box 13145158, Tehran, Iran; 3 Department of Community Nutrition, School of Nutritional Sciences and Dietetics, Tehran University of Medical Sciences, P.O. Box 14155-6117, Tehran, Iran; 4 Pathology and Stem Cell Research Center, Kerman University of Medical Sciences, Kerman, Iran; 5 Cancer Biology Research Center, Cancer Institute of Iran, Tehran University of Medical Sciences, Tehran, Iran; 6 Breast Diseases Research Center, Cancer Institute of Iran, Tehran University of Medical Sciences, Tehran, Iran; 7 Obesity and Eating Habits Research Center, Endocrinology and Metabolism Molecular-Cellular Sciences Institute, Tehran University of Medical Sciences, Tehran, Iran; 8 Department of Community Nutrition, School of Nutrition and Food Science, Isfahan University of Medical Sciences, Isfahan, Iran

**Keywords:** Carbohydrate, Breast neoplasms, Diet, Case-control

## Abstract

**Background:**

A few studies have examined the relationship between carbohydrate quality index (CQI) and risk of breast cancer (BC) among women in Middle Eastern countries. We studied the associations between carbohydrate quality index and the risk of BC in overall and by menopausal status.

**Methods:**

In this case-control study, dietary intake of 461 women with pathologically confirmed BC within the past year were examined. The same information were collected for 495 apparently healthy controls using a 168-item validated FFQ. Carbohydrate quality was determined by considering four criteria including: ratio of solid carbohydrates to total carbohydrates, dietary fiber intake, GI and the ratio of whole grains to total grains.

**Results:**

Mean GI and GL of participants were totally 57.5 ± 7.2 and 245.7 ± 64.7, respectively. A trend toward significant association was seen between GI and odds of BC in the whole population; such that after stratifying analysis by menopausal status, premenopausal women in the highest quartile of GI were 1.85 times higher likely to have BC than those in the lowest quartile (95% CI: 1.12, 3.07, *P* = 0.01). We found that women with the greatest CQI had lower odds for BC, compared with those with the lowest CQI (0.63; 95% CI: 0.43–0.94, *P* = 0.03). This association was remained after stratifying analysis by menopausal status in premenopausal (0.55; 95% CI: 0.34–0.90, *P* = 0.04).

**Conclusion:**

We found that GI was directly and CQI inversely associated with odds of BC. In order to determine the effects of dietary carbohydrate quality prospective cohort studies are needed.

## Introduction

Globally more than 2 million women diagnosed with breast cancer and about 630,000 deaths occurred in 2018, out which half of deaths belongs to low-middle income countries like Iran [1]. The incidence of breast cancer increased among Iranian women in recent years [2]. The age standardized incidence of breast cancer was 31.0 per 100,000 in Iranian women in 2018 [3].

Several risk factors including genetic, lifestyle and dietary intake are related to breast cancer risk [4]. Many studies have evaluated the associations between diets and breast cancer risk, with inconsistent results [5, 6]. High carbohydrate consumption especially refined carbohydrates is one of the important issue in the management of type 2 diabetes mellitus [7]. However, there is growing evidence that intake of high glycemic index diet may also play a crucial role in the etiology of several cancers including breast cancer [8]. This has been hypothesized that diet with a higher glycemic index could increase blood glucose and insulin concentrations which both are related with cancer development [9]. Several factors including amount, type and digestibility of dietary carbohydrate contribute postprandial glycemia and insulin secretion [10]. Hence, this physiologic response can be quantified by the glycemic index (GI) and by the glycemic load (GL). The GI measure carbohydrate quality as it compares the plasma glucose response to specific foods with that induced by the same amount of a standard carbohydrate source (usually white bread or pure glucose) [11]. The GL measure both quantity and quality of dietary carbohydrates [12].

Two case-control studies have illustrated an association between breast cancer risk and dietary glycemic index [13, 14], whereas others have not [15, 16]. The cohort studies reported mixed results about the role of GI/GL on the risk of breast cancer in developed countries [17–21]. Meta-analysis studies on the association between GI and GL and breast cancer risk concluded that there is inconsistency in studies and the proper judgment needs more appropriate studies on this subject [22–24]. According to our knowledge, there was no study with large sample size in Middle East in this regard. Due to different dietary pattern in Middle East, conducting researches to find out this relationship is necessary in this region. However, new studies suggest that GI and GL do not properly assess the carbohydrate quality of diet and introduced other indexes [25].

Previous studies have limitation as the intake of simple components were evaluated with risk of breast cancer, which cannot entirely represent the whole quality of carbohydrate consumption. Therefore using a broader criterion that combine several of these simple components into a compound index which could better represent the whole quality of carbohydrate intake. Some studies defined Carbohydrate Quality Index (CQI) which can take into account dietary fiber intake, GI, whole grains to total grains ratio and solid carbohydrate to total carbohydrates. Only one cohort study on Spanish university graduates women examined the relation between CQI and breast cancer risk [26].

Considering that only one study has been conducted about the association between carbohydrate quality and breast cancer risk and knowing that high consumption of refined carbohydrates (e.g., white rice) are common in the Middle East, [27], this study aimed to examine the relation between GI, GL, carbohydrate intake and CQI with odds of breast cancer among women in a case-control study.

## Subjects and methods

### Participants

We conducted a hospital-based case-control study among Iranian women aged 19–80 years old between 2014 and 2016. The details about this study was described elsewhere [8]. In summary, Cases were patients with pathologically confirmed breast cancer that was diagnosed within the previous year who were admitted at the Cancer Institute of Iran and the controls were healthy visitors of Imam Khomeini Hospital, all controls were matched to cases according to age classification (±10 years) and residential place (Tehran city and non-Tehran city residents). For the current analysis, we excluded 38 participants who had no response to more than 70 items of FFQ and also excluded 35 participants with a total energy intake of more than 5500 or less than 800 kcal/d from the study. After exclusions, the final sample included 461 cases and 495 controls. The study was approved by the Bioethics Committee of Tehran University of Medical Sciences, Tehran, Iran (Ethics code: 93–03–51-27,113).

### Assessment of dietary intake

A 168-item validated food-frequency questionnaire was administered to all patients by trained interviewers. Participants were asked to designate their intake frequency for each food item consumed on a daily, weekly, or monthly basis. Patients reported their consumption over the previous year. Participants who could not respond to their frequency of consumption based on the values mentioned in the questionnaire, had reported their own portion sizes which were converted to the portion size of the questionnaire. The daily portion size of reported consumed foods was calculated and then converted to grams. Total energy intake was calculated by summing up the energy from all foods and nutrient content of foods that were analyzed using the USDA food composition database modified for Iranian foods. In a previous study, the validity and reliability of this FFQ was confirmed by comparing the data from 12 dietary recalls and two similar FFQs that completed 1 year apart.

### Calculation of glycemic index, glycemic load and carbohydrate quality index

Total dietary GI was calculated by using the following formula: Σ (GI_a_ × available carbohydrate_a_)/total available carbohydrate, where GI_a_ is each food’s GI and available carbohydrate was calculated as total carbohydrate_a_ minus fiber_a_. GL values was calculated by multiplying the available carbohydrate content of each food by its GI value and then multiplied the resultant value with the amount of consumption (divided by 100) and then summed the values from all food items. Contribution of four criteria including: ratio of solid carbohydrates to total carbohydrates, dietary fiber intake (g/day), GI and the ratio of whole grains to total grains (whole grains, refined grains and their products) was used to construct CQI. Initially, subjects in each of these four criteria, were divided into quintiles and received a value (ranging from 1 to 5) according to each quintile. But, the scoring of GI was reversed, such that those in the highest quintile were given the score of 1 and those in the lowest quintile were given the score of 5. Finally, the scores were then summed up to compute CQI score (ranging from 4 to 20) [28]. Carbohydrate intake, GI, GL and CQI were energy-adjusted using the residual method and then ranked into quartile.

### Statistical methods

We categorized all participants based on quartiles of Carbohydrate intake, GI, GL and CQI. We used Student t-test and chi-square test to compare the mean of continuous variables and categorical variables of cases and controls, respectively. We applied one-way ANOVA and chi-square test to compare variables across quartiles, where appropriate. We calculated age-, residential place, and energy-adjusted food and nutrient intakes by quartiles of the all criteria using ANCOVA. The association between the dietary Carbohydrate intake, GI, GL and CQI and odds of breast cancer was checked by using crude and multi-variable logistic regression models. The analyses were first adjusted for age and energy (continuous), residential place. Additional adjustments were done for educational level (categorical), parity (nulliparous, 1, 2–3, ≥4), oral contraceptive use (yes vs. no), marital status (married, not married), family history of breast cancer (yes vs. no) body mass index (continuous) and physical activity (continuous) in the second model. All confounders were selected based on recent research. The trend of odds ratios across quartiles of all criteria was examined by considering the median value of criteria in each category as a continuous variable. *P* values < 0.05 were considered statistically significant. The analysis was performed by Stata version 14 (Stata Corp., College Station, TX).

## Results

Compared to controls, cases were slightly older, had lower BMI, were older at first birth, and were more likely to have family history of breast cancer (Table 1). These patients were less likely to be physically active, married, use oral contraceptives, alcohol users and use postmenopausal hormones than controls (Table 1). Women in the top quartile of GI were younger, less likely to be physically active and less alcohol user compared with those in the bottom quartile.

**Table 1:** Baseline characteristics of participants according to the quartiles of glycemic index (GI) and carbohydrate intake

	Case (*n* = 461)	Control (*n* = 495)	*p*-value ^a^	Quartile of glycemic index)units/d)				*p*-value^b^
				1	2	3	4	
No of case/controls				110/129	106/133	122/117	123/116	
Age, year	46.0 ± 10.31	44.05 ± 11.26	0.0003	46.7 ± 10.7	45.9 ± 10.7	43.1 ± 10.8	44.1 ± 10.7	< 0.001
BMI (kg/m2)	28.07 ± 5.20	28.87 ± 6.05	0.01	28.3 ± 4.9	29.0 ± 6.0	28.1 ± 5.9	28.4 ± 5.6	0.47
Physical activity (MET-h/week)	23.09 ± 4.80	29.37 ± 44.19	0.01	34.1 ± 54.3	33.8 ± 51.6	20.5 ± 30.8	16.8 ± 22.2	< 0.001
Age at menarche (years)	13.02 ± 2.54	12.92 ± 2.76	0.28	12.9 ± 2.6	13.1 ± 2.4	12.7 ± 2.7	13.0 ± 2.7	0.50
Age at first birth (years)	18.89 ± 8.51	17.3 ± 8.27	0.002	18.5 ± 8.42	17.8 ± 8.1	17.8 ± 8.6	18.0 ± 8.5	0.78
Menopausal status (%)								
Premenopausal	301(66.59)	325 (67.15)	0.85	133 (56.6)	157 (66.8)	170 (72.9)	165 (70.5)	0.01
Postmenopausal	151(33.41)	159 (32.85)		102 (43.4)	78 (33.1)	63 (27.0)	69 (29.4)	
Educational level (%)								
Un university	379 (83.85)	406 (84.06)	0.93	191 (81.6)	203 (8.3)	190 (81.9)	201 (85.9)	0.34
University	73 (16.15)	77 (15.94)		43 (18.3)	32 (13.6)	42 (18.1)	33 (14.1)	
Marital status (%)								
Married	369 (81.6)	415 (87.0)	0.05	195 (83.6)	197 (84.9)	196 (84.8)	196 (84.1)	0.97
Unmarried/divorced/widowed	83 (18.3)	62 (13.0)		38 (16.3)	35 (15.0)	35 (15.1)	37 (15.8)	
Family history of breast cancer (%)	44 (9.73)	7 (1.42)	0.000	13 (5.4)	12 (5.0)	13 (5.5)	13 (5.4)	0.99
Oral contraceptive use (%)	236 (53.03)	258 (61.43)	0.01	117 (53.9)	135 (62.7)	116 (54.9)	126 (56.7)	0.24
Current smoker (%)	16 (3.54)	24 (4.98)	0.27	14 (5.9)	5 (2.1)	8 (3.4)	13 (5.5)	0.12
Alcohol use (%)	12 (2.65)	29 (6.00)	0.01	15 (6.3)	7 (2.9)	14 (6.0)	5 (2.1)	0.05
Postmenopausal hormone use (%)	2 (0.43)	10 (2.02)	0.02	3 (1.2)	4 (1.6)	1 (0.4)	4 (1.6)	0.56
Parity								
Nulliparous/missing	210 (42.42)	204 (44.25)	0.75	109 (45.6)	97 (40.5)	109 (45.6)	99 (41.4)	0.33
1	51(10.30)	39 (8.46)		27 (11.3)	15 (6.2)	22 (9.2)	26 (10.8)	
2–3	154 (31.11)	147 (31.89)		69 (28.8)	79 (33.0)	72 (30.1)	81 (33.8)	
≥ 4	80 (16.16)	71 (15.40)		34 (14.2)	48 (20.0)	36 (15.0)	33 (13.8)	

^a^χ^2^ Test for ordinal qualitative variables and t-test for continuous variable

^b^*P*-values were determined by the ANOVA test

The mean GI of all participants was 57.5 (± 7.2) and no significant differences were seen between cases and controls. The mean (± SD) of CQI was 11.5 (± 3.4) in all participants the comparison of CQI indicated no different between cases and controls. Subjects in the top quartile of GI had lower intakes of total carbohydrate, solid carbohydrate, fiber, protein, fruits, vegetables, high fat dairy, low fat dairy, legumes, vitamin C, D, B6, B9, and B12 than those in the lowest quartile (Table 2).

**Table 2:** Baseline nutrient and food group intake by quartiles of energy-adjusted glycemic index (GI) of participants

	quartiles of energy-adjusted glycemic index				
	1	2	3	4	*P*-value ^a^
Dietary GI (units/day)	48.5 ± 3.2	54.8 ± 1.3	59.7 ± 1.4	66.8 ± 3.7	< 0.001
Dietary GL (units/day)	215.7 ± 60.0	245.6 ± 55.5	248.7 ± 56.1	274.3 ± 73.2	< 0.001
Total carbohydrate (g/day)	442.3 ± 114.2	447.2 ± 102.4	416.0 ± 93.8	411.4 ± 107.0	< 0.001
Solid carbohydrates (g/day)	427.8 ± 169.4	429.6 ± 167.4	405.4 ± 148.3	389.5 ± 162.5	0.01
Liquid carbohydrates (g/day)	17.0 ± 21.2	17.7 ± 21.5	15.0 ± 21.1	41.8 ± 19.0	0.32
CQI scores	15.2 ± 2.3	13.2 ± 2.9	11.5 ± 2.5	8.7 ± 2.4	< 0.001
Energy (kcal/day)	2815.8 ± 1102.5	2796.9 ± 1017.5	2830.9 ± 1023.9	2739.7 ± 1076.5	0.79
Fiber (g/day)	47.2 ± 13.8	42.0 ± 12.3	33.8 ± 9.5	27.7 ± 9.1	< 0.001
Protein (g/day)	94.0 ± 36.1	83.6 ± 21.8	78.7 ± 22.9	75.5 ± 22.4	< 0.001
Fat (g/day)	126.8 ± 40.7	119.4 ± 38.1	125.4 ± 38.9	120.7 ± 43.8	0.13
Saturated fats (g/day)	49.8 ± 24.2	47.0 ± 21.1	50.0 ± 21.9	48.6 ± 23.8	0.46
Fruits (g/day)	828.9 ± 450.4	661.2 ± 327.5	501.2 ± 254.5	358.8 ± 218.1	< 0.001
Vegetables (g/day)	479.0 ± 273.6	381.5 ± 222.1	305.0 ± 176.7	257.6 ± 154.0	< 0.001
Red meat (g/day)	15.0 ± 14.0	16.2 ± 31.3	14.9 ± 20.6	15.8 ± 26.3	0.87
High fat dairy (g/day)	248.6 ± 165.1	222.5 ± 153.1	210.5 ± 172.4	169.0 ± 144.7	< 0.001
Low fat dairy (g/day)	121.4 ± 216.7	54.7 ± 102.1	47.8 ± 87.4	40.8 ± 90.9	< 0.001
Potatoes (g/day)	20.4 ± 25.0	24.2 ± 51.2	25.0 ± 24.2	25.0 ± 24.2	0.59
Rice (g/day)	105.9 ± 79.9	167.7 ± 90.8	221.5 ± 108.9	351.3 ± 170.4	< 0.001
Sugar (g/day)	26.3 ± 28.5	29.2 ± 23.3	31.4 ± 25.0	31.8 ± 24.8	0.07
Soft drinks (g/day)	56.5 ± 112.0	84.1 ± 143.0	68.1 ± 162.6	80.2 ± 128.0	0.12
Sweet desert (g/day)	10.6 ± 14.1	13.8 ± 21.2	12.4 ± 20.8	10.4 ± 19.4	0.17
Legumes (g/day)	71.4 ± 89.4	53.6 ± 82.5	40.6 ± 33.9	36.2 ± 32.8	< 0.001
Vitamin B6 (mg/d)	2.9 ± 0.94	2.4 ± 0.73	2.0 ± 0.55	1.7 ± 0.55	< 0.001
Folate (mcg/d)	478.9 ± 170.0	406.4 ± 148.8	338.7 ± 90.2	290.0 ± 90.7	< 0.001
Vitamin B12 (mcg/d)	6.07 ± 4.1	4.9 ± 3.4	4.0 ± 2.3	3.4 ± 2.2	< 0.001
Vitamin C (mcg/d)	376.8 ± 165.8	307.4 ± 147.2	233.7 ± 92.0	179.6 ± 90.5	< 0.001
Vitamin D (mcg/d)	3.9 ± 4.0	2.8 ± 3.2	2.5 ± 2.4	2.3 ± 2.6	< 0.001

^a^All value were adjusted for energy, except for dietary energy intake, by using ANCOVA

Multivariable-adjusted odds ratios and 95% CIs for breast cancer, separately for whole population, pre- and post-menopausal women, across quartile categories of carbohydrate, glycemic index, glycemic load and carbohydrate quality index are provided in Table 3**.** No significant association was observed between carbohydrate intakes and glycemic load with odds of breast cancer in the whole study population. However, a trend toward significant association was seen between GI and odds of breast cancer in the whole population, such that after controlling for several potential confounders, individuals in the highest quartile of GI were 1.41 times more likely to have breast cancer than those in the lowest quartile (OR: 1.41; 95% CI: 0.96–2.07; *P* = 0.04). Women in the highest quartile of CQI had significantly lower risk of breast cancer than those in the lowest quartile (OR: 0.63, 95% CI: 0.43–0.94; *P* = 0.03). When stratified by menopausal status, we found that premenopausal women in the top category of GI had higher odds of breast cancer, compared with those in the bottom category (OR: 1.85; 95% CI: 1.12–3.07; *P* = 0.01). In addition, premenopausal women in the top category of CQI had lower odds of breast cancer compared with those in the bottom category (OR: 0.55; 95% CI: 0.34–0.90; *P* = 0.04). We found no significant association between all carbohydrate quality criteria (i.e. GI, GL, and CQI) and odds of breast cancer in postmenopausal women, either before or after controlling for confounders.

**Table 3:** Risk for breast cancer according to quartiles of the Carbohydrate, glycemic index (GI), glycemic load (GL) dietary and carbohydrate quality index (CQI)

	OR (95% CI)				*P*_Trend_^a^
	Quartile 1	Quartile 2	Quartile 3	Quartile 4	
Total					
Carbohydrate					
No.of cases/controls (461/495)	121/118	119/120	114/125	107/132	
Crude	1	0.96 (0.67–1.38)	0.88 (0.62–1.27)	0.79 (0.55–1.13)	0.17
Model 1^b^	1	1.05 (0.72–1.53)	0.91 (0.63–1.34)	0.75 (0.52–1.08)	0.10
Model 2^c^	1	0.97 (0.65–1.44)	0.85 (0.58–1.25)	0.74 (0.50–1.08)	0.09
Glycemic index					
No.of cases/controls (461/495)	110/128	106/133	127/117	123/116	
Crude	1	0.93 (0.65–1.34)	1.22 (0.85–1.75)	1.24 (0.86–1.76)	0.11
Model 1	1	0.95 (0.65–1.39)	1.33 (0.91–1.92)	**1.39 (0.96–2.01)**	0.02
Model 2	1	1.04 (0.69–1.54)	1.34 (0.91–1.97)	**1.41 (0.96–2.07)**	0.04
Glycemic load					
No.of cases/controls (452/494)	116/123	114/125	115/124	116/123	
Crude	1	0.96 (0.67–1.38)	0.98(0.68–1.40)	1.00 (0.69–1.43)	0.97
Model 1	1	1.02 (0.70–1.50)	1.01 (0.69–1.48)	0.96 (0.67–1.39)	0.85
Model 2	1	0.95 (0.64–1.42)	0.91 (0.61–1.34)	0.94 (0.64–1.37)	0.71
CQI					
No.of cases/controls (452/494)	126/113	117/122	113/126	105/134	g
Crude	1	0.82 (0.58–1.18)	0.87 (0.61–1.24)	0.69 (0.48–0.99)	0.07
Model 1	1	0.81 (0.56–1.16)	0.81 (0.56–1.15)	0.61 (0.42–0.89)	**0.01**
Model 2		0.86 (0.59–1.25)	0.85 (0.59–1.24)	**0.63 (0.43–0.94)**	**0.03**
Premenopausal					
Carbohydrate					
No.of cases/controls (300/325)	75/82	80/76	79/77	66/90	
Crude	1	1.09 (0.70–1.69)	1.02 (0.65–1.59)	0.87 (0.55–1.36)	0.51
Model 1	1	1.24 (0.77–2.00)	1.05 (0.66–1.67)	0.82 (0.52–1.31)	0.33
Model 2		1.12 (0.68–1.84)	0.98 (0.61–1.59)	0.82 (0.51–1.33)	0.37
Glycemic index					
No.of cases/controls (300/325)	66/91	74/82	77/79	83/73	
Crude	1	1.21 (0.75–1.98)	1.47 (0.92–2.34)	**1.63 (1.02–2.60)**	**0.02**
Model 1	1	1.21 (0.74–1.98)	1.58 (0.98–2.53)	**1.71 (1.06–2.74)**	**0.01**
Model 2		1.35 (0.80–2.26)	1.62 (0.98–2.67)	**1.85 (1.12–3.07)**	**0.01**
Glycemic load					
No.of cases/controls (300/325)	76/81	71/85	74/82	79/77	
Crude	1	0.99 (0.62–1.56)	0.97 (0.62–1.51)	1.12 (0.72–1.75)	0.63
Model 1	1	1.11 (0.68–1.81)	1.04 (0.65–1.65)	1.13 (0.72–1.77)	0.67
Model 2		1.04 (0.62–1.73)	0.92 (0.57–1.49)	1.14 (0.71–1.81)	0.70
CQI					
No.of cases/controls (300/325)	81/76	76/80	79/77	64/92	
Crude	1	0.89 (0.57–1.38)	0.95 (0.61–1.47)	0.68 (0.43–1.06)	0.14
Model 1	1	0.88 (0.56–1.38)	0.92 (0.59–1.44)	**0.57 (0.36–0.91)**	**0.03**
Model 2	1	0.84 (0.53–1.35)	0.94 (0.59–1.49)	**0.55 (0.34–0.90)**	**0.04**
Postmenopausal					
Carbohydrate					
No.of cases/controls (152/160)	44/34	37/41	32/46	39/39	
Crude	1	0.75 (0.40–1.39)	0.54 (0.28–1.03)	0.77 (0.42–1.41)	0.28
Model 1	1	0.69 (0.36–1.31)	**0.51 (0.26–0.98)**	0.79 (0.43–1.45)	0.32
Model 2		0.63 (0.32–1.24)	**0.40 (0.19–0.82)**	0.77 (0.41–1.46)	0.25
Glycemic index					
No.of cases/controls (152/160)	39/39	37/41	36/42	40/38	
Crude	1	0.80 (0.42–1.52)	0.80 (0.42–1.52)	1.04 (0.56–1.91)	0.86
Model 1	1	0.83 (0.43–1.58)	0.83 (0.43–1.59)	1.05 (0.57–1.94)	0.82
Model 2	1	0.89 (0.45–1.75)	0.86 (0.43–1.71)	1.01 (0.53–1.91)	0.97
Glycemic load					
No.of cases/controls (152/160)	41/37	40/38	37/41	34/44	
crude	1	0.85 (0.46–1.60)	0.88 (0.47–1.64)	0.79 (0.42–1.48)	0.50
Model 1	1	0.82 (0.43–1.57)	0.89 (0.46–1.70)	0.78 (0.41–1.46)	0.51
Model 2	1	0.74 (0.37–1.47)	0.78 (0.39–1.54)	0.73 (0.38–1.40)	0.40
CQI					
No.of cases/controls (152/160)	43/35	40/38	31/47	38/40	
Crude	1	0.95 (0.51–1.77)	0.52 (0.26–1.02)	0.82 (0.45–1.51)	0.28
Model 1	1	0.96 (0.51–1.78)	0.52 (0.26–1.02)	0.83 (0.45–1.53)	0.29
Model 2		1.03 (0.53–1.99)	0.59 (0.29–1.20)	0.87 (0.46–1.66)	0.41

^a^Trend based on median values of each quartile

^b^Model 1: Adjusted for age, energy and residential place

^c^Model 2: further adjusted for oral contraceptive use, parity, marital status, family history of breast cancer, BMI (continuous), and physical activity

## Discussion

In this study we observed a significant association of high GI with odds of breast cancer in the whole population. Regarding CQI the results were reversed that those women with higher CQI (quartile 4 of the score) had a significantly lower odds of breast cancer compared with those who had the lower CQI (quartile 1). Also, we found the same results for GI and CQI in the premenopausal women. To our knowledge, this case-control study is among the first investigations that reports the association between Total carbohydrates, GI, GL and CQI with odds of breast cancer with large sample size in a Middle Eastern country.

This study examined several dietary carbohydrates that may have varied effect on blood glucose responses. The association of this criteria with risk of breast cancer have been frequently assessed by several studies with inconsistent results. Previously a study showed that 60% of total energy intake was supplied by carbohydrates among Iranian [29] especially refined grain products [27] which can result in high GI. GI and GL are measures that evaluate different aspects of total carbohydrate intake. The GI represents a food’s relative postprandial blood glucose spike and the GL measures the product of GI and the amount of dietary total carbohydrate. Barclay et al. conducted a meta-analysis on prospective cohort studies for assessing the relationships between GI and GL with breast cancer risk and showed an 8% increase in the risk of breast cancer for high GI [30]. We observed no overall association between GL and odds of breast cancer, also after stratifying by menopausal status. In our study, subjects with high GL did not have higher intake of red meat or processed meat which was associated with risk of breast cancer (results not shown). Our results are consistent with a meta-analysis of 10 cohort study that illustrated no significant association between dietary GL and breast cancer risk [23].

We found a significant association between dietary GI and risk of breast cancer among whole participants and premenopausal women. In line with our results Alboghobeish et al. found an association between high GI and risk of breast cancer [14]. However, Dietary carbohydrate intake, GL and GI were not related to risk of breast cancer in a large European cohort study [31]. The same results was found in the Women’s Health Initiative study [32]. In the analyzing of EPIC-Italy study after exclude subjects who had dieting at recruitment found that high GL was significantly associated increased breast cancer risk [33]. Canadian women survey showed consumption of the diets with high GI may be related with increased risk of breast cancer among postmenopausal women [17]. While, dietary carbohydrate intake, GL, and GI were not related to risk of breast cancer among premenopausal women of Nurses’ Health Study [34]. Nevertheless, reducing intake of high GI and GL foods, like refined carbohydrates, may offer a benefit in preventing of breast cancer risk [23]. In this regard, the suggested mechanisms is high insulinemia that resulted in high glycemic diets, may inhibit apoptosis and synthesis of IGF binding proteins which promotes cellular proliferation [20].

In this study we observed all participants and premenopausal with best CQI had a significantly lower risk of breast cancer. The total quality and quantity of carbohydrate and their dietary source are related to cancers. Recent discussion indicate that fiber and whole grain should be considered for carbohydrate quality as well as GI [25]. In addition the potential resultant effects of solid and liquid carbohydrate is not similar [35]. Therefore, the CQI that consider fiber, GI, solid or liquid form of carbohydrate and whole or refined grains seems to be better index for assessment. Similar to our study, the sun project cohort study observed that a higher CQI was associated with a lower risk of breast cancer and after stratifying by menopausal status, found an inverse association between top quartile of CQI comparison of lower quartile among premenopausal women [26]. Other previous studies examined the relation between components of CQI with breast cancer, as alone. A meta-analyses of 16 prospective studies showed a 5% reduction in breast cancer risk by extracting the risk estimate of the highest versus the lowest intake of dietary fiber [36]. A high fiber diet provides many health benefits including weight loss, lower cholesterol levels, and decrease insulin resistance. In addition fiber can reduce circulating estrogen levels by changing the gut microbiota which affect the reactivation of conjugated estrogens [37]. Reabsorption of deconjugated estrogen influence estrogen metabolism which is related with hormone dependent cancers, such as breast cancer [38]. Also, the role of other dietary component of CQI in decreasing risk of cancers via reducing levels of inflammatory markers have been assessed. Whole grains are rich in antioxidants which are major elements of antioxidants enzymes activities and have been inversely linked to breast cancer risk. So, whole grains have phytoestrogens and polyphenols which have antioxidant properties and potential to inhibit cell multiplication and angiogenesis and to consequence cell apoptosis [39].

The strengths of the current study include considering several potential confounders, the use of validated questionnaires for dietary assessment, recruiting participants from a referral hospital, in which subjects are from the whole country. Stratified analysis by menopausal status is strength of this study. However, several limitations need to be considered. First, due to the case-control design of the study with its inherent recall and selection bias, one cannot infer causality. Second, misclassification of participants in terms of dietary intakes cannot be excluded due to the use of FFQ in the current study. Third, we did not have information of the hormone receptor status of the tumor for the participants.

## Conclusion

In conclusion, we found a significant association between carbohydrate quality and breast cancer. So considering the carbohydrate type intake might be important for cancer control in society. Additional studies are required to prospectively examine the association of carbohydrate quality and risk of breast cancer considering the specific subgroups of estrogen receptor.

## Data Availability

The datasets used and/or analyzed during the current study are available from the corresponding author on reasonable request.
